# Efficient anaerobic sediment processing via a novel sediment core extruder

**DOI:** 10.1016/j.mex.2022.101664

**Published:** 2022-03-28

**Authors:** Matthew Quinan, Csaba Vazco, Jordon Beckler

**Affiliations:** Harbor Branch Oceanographic Institute, Florida Atlantic University, USA

**Keywords:** Engineering, Sediment core extrusion, Geochemistry, Lake Okeechobee, Redox speciation

## Abstract

Collection, extrusion and sampling of sediment cores are common methods used to explore the biogeochemistry of marine and lake deposits. We present a novel design for a sediment-core extruder that has greatly improved our core processing efficiency and reduced costs, while ensuring reliable results from solid phase and porewater analyses. The free-standing, aluminum structure is lightweight, enabling easy transport and quick setup/breakdown when processing cores in the field, and the height of the apparatus can be adjusted to fit any lab space and create a comfortable sampling experience for the user. Set up and processing time are reduced by eliminating the need for clamps or collars that secure the core barrel. A unique feature is the novel means to attach a portable glove bag for anaerobic sample processing. Dissolved iron speciation data from sediment cores collected in hypereutrophic Lake Okeechobee, Florida, USA, are presented to demonstrate the utility of the device in maintaining redox conditions throughout the process of core extrusion and sectioning.

• A sediment extrusion device is described to allow efficient sediment core processing (separation) for subsequent biogeochemical analyses
• The extruder is portable and can be adapted to cores of any diameter.
• The extruder can be outfitted with an optional novel mechanism for attaching a glove bag to allow for anaerobic processing, and testing demonstrates that reduced iron is maintained in the dissolved state.

## Graphical abstract

**Figure:**
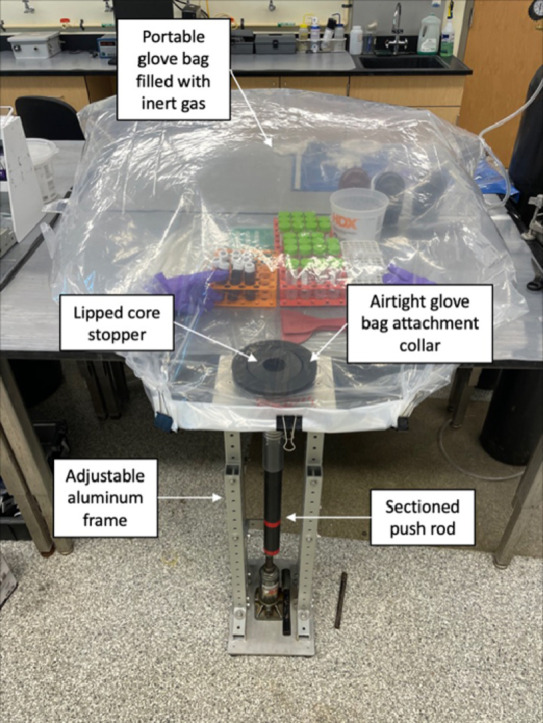

Specifications table

**Table:** 

Subject Area:	Earth and Planetary Sciences
More specific subject area:	*Sediment biogeochemistry*
Method name:	Sediment core extrusion
Name and reference of original method:	*NA*
Resource availability:	Engineering files available upon request from authors

## Background

Collection and sectioning (or “slicing”) of sediment cores at fine-scale increments has for decades been common practice in exploring the biogeochemistry of marine and lacustrine ecosystems [1], [2], [3], [4], [5], [6], [7], [8]. The process, however, can be onerous, and the often cramped or makeshift laboratory conditions typical of oceanographic research vessels and other sampling sites increase the difficulty of this task. Improvements have been made throughout the years, many involving specially constructed apparatuses that both expedite the process and ensure accurate and repeatable results from subsequent solid phase and pore water analyses [9], [10], [11]. We recently developed, and now routinely employ, a novel apparatus to extrude and section sediment cores of different sizes. This has greatly increased our sampling efficiency while helping to maintain the redox speciation of samples prior to analysis.

**Fig. 1:**
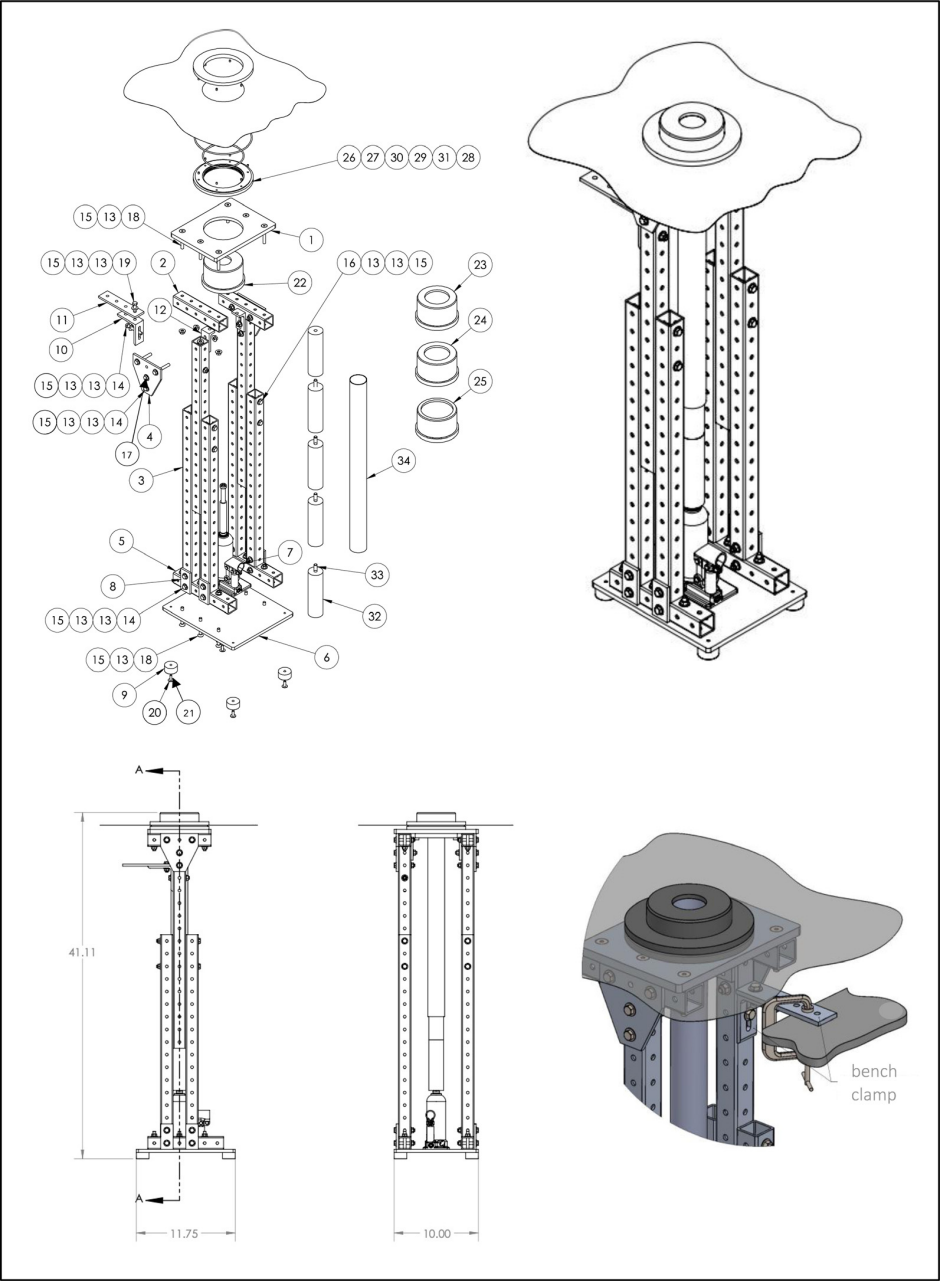
Extruder design with item no. identifiers (correspond to Table 1).

The apparatus (Fig. 1) was constructed mostly of commercially available parts totalling < $600 (Table 1). However, approximately 30–40 h of custom machining may bring the total cost closer to $2500. Sectioning of sediment cores is often carried out near the coring location, immediately after collection, to reduce the risk of sampling/processing artifacts, e.g., sediment mixing and oxidation. Our extruder enables sectioning of cores of three diameters (2-, 3-, and 4-inch) in rapid succession, although stoppers can be created to accommodate cores of any diameter. The lightweight, free-standing structure enables easy transport, and quick assembly in a new lab space. To accommodate both changes in processing location and the individual who sections the core, the height of the apparatus can be adjusted easily, increasing its usability and resulting in a more comfortable sampling experience.

**Table 1:** List of parts and costs. “HBOI” as the vendor indicates custom components designed at FAU Harbor Branch.

Item No.	Part No.	Description	Qty.	Vendor	Vendor of Base Part	Vendor No. of Base Part	Part Cost	Total Cost
**1**	100–004–060	TOP PLATE, SAMPLE EXTRACTION PRESS, SEDIMENT CORER	1	HBOI			$15.00	$15.00
**2**	100–004–064	TUBE, AL, 1.500 SQ RAIL BOLTED FRAMING, 7.500 L	2	HBOI	McMaster-Carr	8809T7	$54.22/10 ft	$3.44
**3**	100–003–349	TUBE, AL, 1.500 SQ RAIL BOLTED FRAMING, 24.000 L	6	McMaster-Carr			$54.22/10 ft	$66.24
**4**	100–004–054	TEE SURF BRKT, AL, 1.500 SQ RAIL T-SLOT FRAMING 4.500 L	2	McMaster-Carr			$8.16	$16.32
**5**	100–004–071	TUBE, AL, 1.500 SQ RAIL BOLTED FRAMING, 9.000 L	2	HBOI	McMaster-Carr	8809T7	$54.22/10 ft	$6.90
**6**	100–004–057	BASE PLATE, SAMPLE EXTRACTION PRESS, SEDIMENT CORER	1	HBOI			$15.00	$15.00
**7**	100–004–061	HYDRAULIC BOTTLE JACK, MANUAL, 6.625 MAX LIFT	1	Any vendor			$25.00	$25.00
**8**	100–004–062	STRAIGHT SURF BRKT, AL, 1.500 SQ RAIL T-SLOT FRAMING, 3.000 L	4	McMaster-Carr			$6.39	$25.56
**9**	100–004–243	UNTHREADED BUMPER, SBR, SHORE A 70, ID 0.250, CB 0.750, OD 1.500, 0.750 H	4	McMaster-Carr			$8.87/10 count	$3.55
**10**	100–004–077	MODIFIED CORNER BRKT, AL, SILVER, 1.500 SQ RAIL T-SLOT FRAMING, 3.000 L	1	HBOI	McMaster-Carr	47065T241	$6.26	$6.26
**11**	100–003–569	STRAIGHT SURF BRKT, AL, SILVER, 1.500 SQ RAIL BOLTED FRAMING, 6.000 L	1	McMaster-Carr			$6.66	$6.66
**12**	100–004–078	TUBE STOPPER HOLDER, SAMPLE EXTRACTION PRESS, SEDIMENT CORER	2	HBOI	McMaster-Carr	47065T241	$6.26	$12.52
**13**	100–000–024	FW, G P, SS316, SIZE 0.313, ID 0.344, OD 0.750, 0.043 THK	56	McMaster-Carr			$9.15/100 count	$5.12
**14**	100–000–806	HHCS, SS316, 0.313–18 UNC - 2A, 2.250 L, MIN THRD L 0.875	15	McMaster-Carr			$3.47/5 count	$10.41
**15**	100–000–228	NUT, HEX, SS316, 0.313–18 UNC - 2B, 0.500 W, 0.266 THK	34	McMaster-Carr			$5.42/50 count	$3.69
**16**	100–000–821	HHCS, SS316, 0.313–18 UNC - 2A, 5.000 L, MIN THRD L 0.875	4	McMaster-Carr			$1.07	$4.28
**17**	100–000–808	HHCS, SS316, 0.313–18 UNC - 2A, 2.500 L, MIN THRD L 0.875	2	McMaster-Carr			$3.42/5 count	$1.37
**18**	100–001–340	FHSCS, HEX, SS316, 0.313–18 UNC - 3A, 2.500 L, MIN THRD L 1.125	12	McMaster-Carr			$1.70	$20.40
**19**	100–000–796	HHCS, SS316, 0.313–18 UNC - 2A, 1.000 L, FULL THRD	1	McMaster-Carr			$3.69/10 count	$0.37
**20**	100–000–023	FW, G P, SS316, SIZE 0.250, ID 0.281, OD 0.625, 0.043 THK	4	McMaster-Carr			$7.11/100 count	$0.28
**21**	100–004–170	BHCS, HEX, SS316, 0.250–20 UNC - 3A, 0.750 L, FULL THRD	4	McMaster-Carr			$10.10/25 count	$1.62
**22**	100–004–068	STOPPER, 2.000 IN TUBE, SAMPLE EXTRACTION PRESS, SEDIMENT CORER	1	HBOI	McMaster-Carr	8576K38	$45.90	$45.90
**23**	100–004–069	STOPPER, 3.000 IN TUBE, SAMPLE EXTRACTION PRESS, SEDIMENT CORER	1	HBOI	McMaster-Carr	8576K38	$45.90	$45.90
**24**	100–004–070	STOPPER, 3.500 IN TUBE, SAMPLE EXTRACTION PRESS, SEDIMENT CORER	1	HBOI	McMaster-Carr	8576K39	$45.90	$45.90
**25**	100–004–074	STOPPER, 4.000 IN TUBE, SAMPLE EXTRACTION PRESS, SEDIMENT CORER	1	HBOI	McMaster-Carr	8576K38	$45.90	$45.90
**26**	100–004–067	GLOVE BAG SEAL BOTTOM, SAMPLE EXTRACTION PRESS, SEDIMENT CORER	1	HBOI	McMaster-Carr	9986K71	$28.79	$28.79
**27**	100–004–073	MAGNET, NEODYMIUM, MAG DIR THRU THKNS, 0.188 OD, 0.188 THK	12	McMaster-Carr			$0.71	$8.52
**28**	100–004–072	O-RING, BUNA-N, SHORE A 70, 2–260	1	Any vendor			$9.99/10 count	$1.00
**29**	100–004–276	O-RING, BUNA-N, SHORE A 70, 2–247	1	Any Vendor			$9.99/25 count	$0.40
**30**	100–004–067	GLOVE BAG SEAL TOP, SAMPLE EXTRACTION PRESS, SEDIMENT CORER	1	HBOI	McMaster—Carr	9986K71	$28.79	$28.79
**31**	Glove bag		1	Any Vendor			$30.00	$30.00
**32**	100–004–063	PUSH ROD, SAMPLE EXTRACTION PRESS, SEDIMENT CORER	5	HBOI	McMaster-Carr	8497K413	$12.91/1 ft	$38.73
**33**	100–004–079	DOWEL, STEEL ALLOY, 3/8″ DIA, 1 7/8″ L	4	McMaster-Carr			$4.00	$16.00
**34**	100–003–565	BARREL, ACRYLIC, OD 2.000, ID 1.875, SEDIMENT CORER	1	HBOI			$13.00	$13.00
**35**	100–005–002	PISTON PLUG, CUT LDPE TUBING, DIAMETER SHOWN FOR 2″ CORE	Any	HBOI	McMaster-Carr	8624K18	$22.36/ft	$22.36
**36**	100–005–003	O-RING, BUNA-N, SHORE A 70, 2–223	2 per plug	McMaster—Carr	McMaster-Carr	9452K61	$15.16/100 count	$15.16
								**$636.34**

Cost of parts: $636.34.

Engineer (40 h): $1600.00.

Total cost: $2236.34.

When sampling for redox-sensitive analytes, oxidation artifacts can manifest rapidly when suboxic or anoxic sediment/soil sections are exposed to oxic conditions [12]. A glove bag filled with an inert gas, e.g., N_2_, is often fitted over the exposed sediment core to create an artificial anoxic environment for sectioning. Similar to several other geochemistry labs’ procedures (based on our collaborations), we previously used electrical tape to attach and seal the open end of a standard two-hand glove bag to the upper portion of the core barrel prior to purging the ambient air containing oxygen. This setup was prone to leaks, awkward to assemble, and made it difficult to ensure good ergodynamics with respect to positioning of the hands relative to the core, i.e. so as to not restrict movement. The new extruder is fitted with a two-piece collar that clasps together magnetically on either side of a hole cut in the glove bag, forming an air-tight seal around the core. We are excited to share our novel extruder design with the broader community, and we are confident they will find that it makes anaerobic sediment core sectioning more efficient and easier, while reducing costs and ensuring mobility.

## Method

Once assembled, the height of the apparatus frame (Fig. 3A) can be adjusted by removing the bolts (Fig. 1, Item No. 16, 13, 15) from the legs on either side of the apparatus (Fig. 1- Item No. 4), lifting the top of the structure to the desired height, and re-inserting and securing the bolts. This is done to accommodate the lab space in which the core is being sectioned, the individual sectioning the core, and the length of the core being sectioned (maximum core length of 2.75 feet). The apparatus can then be placed wherever extrusion and sampling will be undertaken. The rubber feet (Fig. 1- Item No. 9) ensure that the apparatus does not move, regardless of the surface on which it is placed. The apparatus can be further fastened in place by clamping the corner bracket (Fig. 1- Item No. 10, 11) protruding from apparatus to a benchtop.

**Fig. 2:**
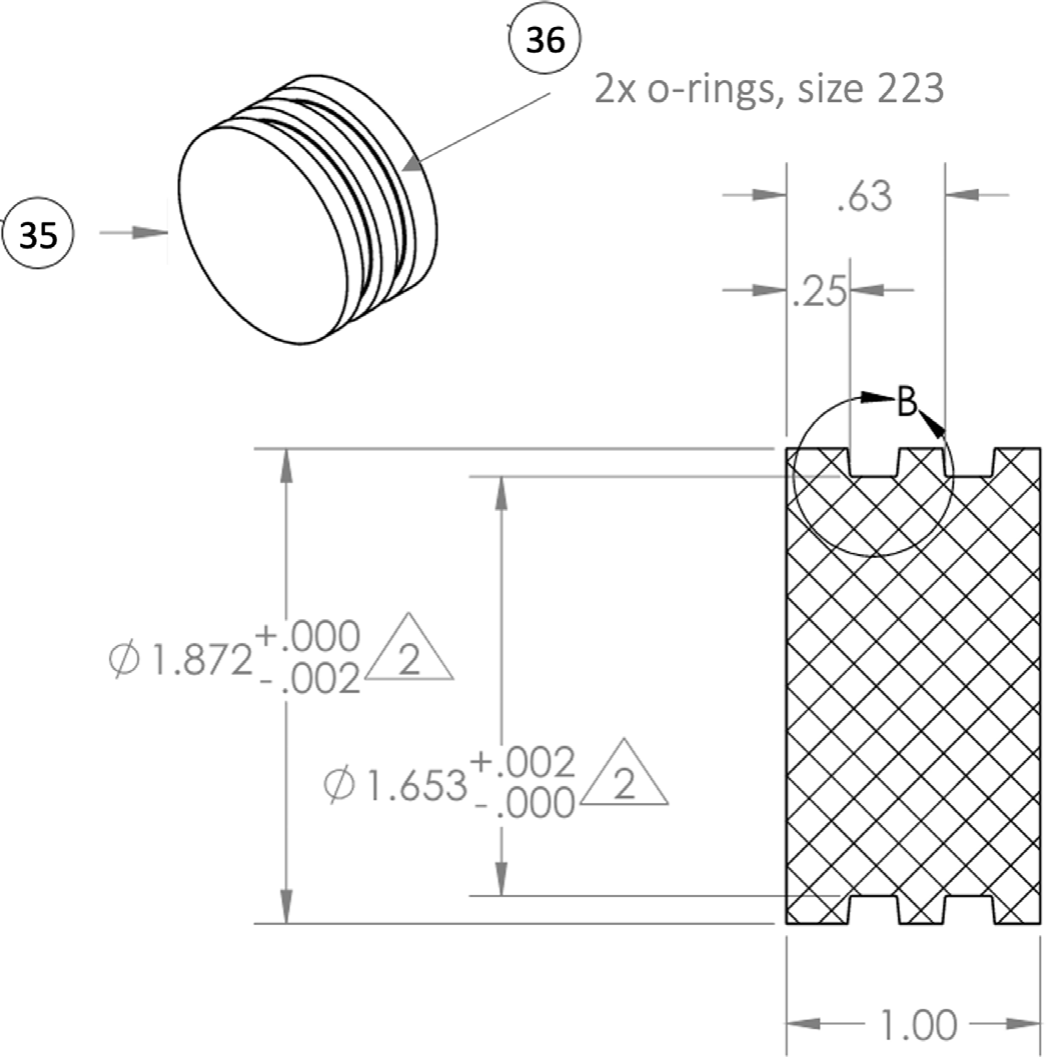
Example piston plug for 1.875″ inner diameter cores with no. identifiers (correspond to Table 1).

**Fig. 3:**
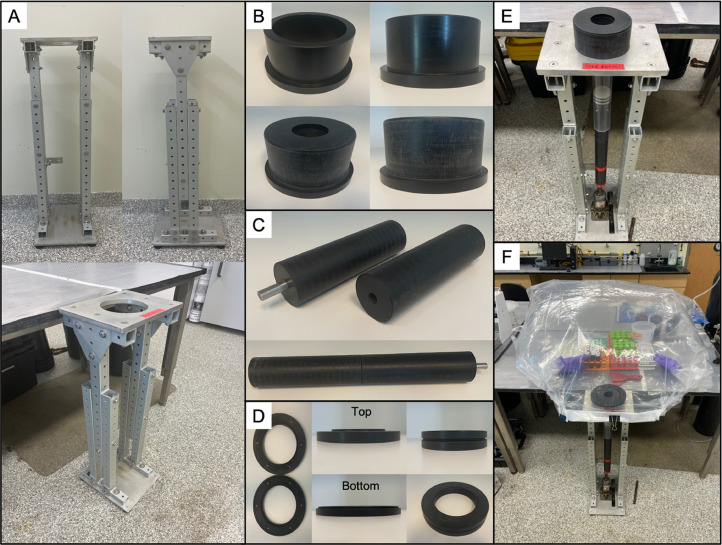
(A) Adjustable core extruder apparatus frame. (B) Lipped stopper for holding barrel (4-inch and 2-inch) in place during sectioning (the lip is not visible as it is on the inside top edge of the stopper). (C) Push rod sections. (D) Glove bag collar assembly. (E) Core extruder with stopper protruding from the top plate and piston plug visible inside core. (F) Core extruder with glove bag attached using the glove bag collar for anaerobic core sectioning.

**Fig. 4:**
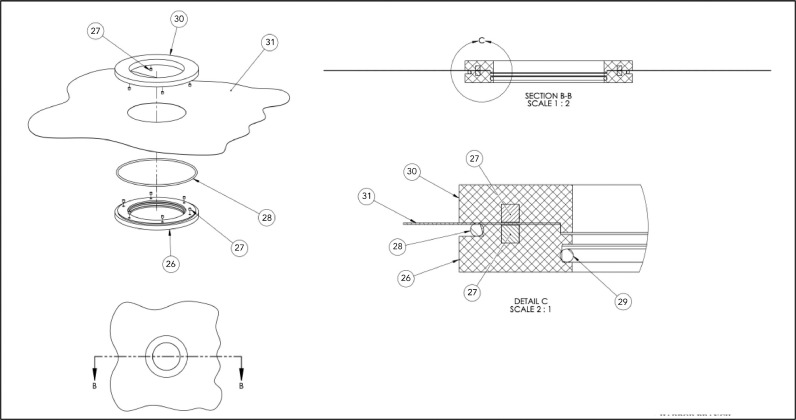
Glove bag collar assembly with no. identifiers (correspond to Table 1).

Following the collection of a sediment core, caps are placed on both ends of the core barrel (Fig. 1- Item No. 34) to prevent the loss of sediment and overlying water. Rapid, efficient sampling is important at this stage, as an extended period of sealing can promote anoxia in the overlying water and the artifactual mobilization of reduced ions and other analytes (e.g., phosphate) from sediment pore waters into the overlying water. In preparation for core extrusion, the bottom cap is removed, but the top cap is left in place to create a vacuum and immobilize the sediment. Next, a piston plug with a diameter that matches the inner diameter of the core barrel (Fig. 2) is inserted into the bottom of the core barrel in one single motion while the top cap is removed. The piston plugs are cut from an LDPE rod, lathed to the correct diameter, and grooves cut for o-rings. This extruder piston enables the sediment to be pushed upward and out the top of the core barrel in successive increments (known colloquially as “sectioning”) while preserving the core stratigraphy. The “plugged” sediment core is then inserted into the appropriate removable stopper (Fig. 1- Item No. 22, 23, 24, 25, Fig. 3B) with a matching inner diameter, machined on a circular lathe. Importantly, a few cm of overlying water should be left intact during this process to ensure that the surface sediment is not exposed to air during glove bag filling. In Fig. 3B, stoppers for both 2″ and 4″ cores are shown; notice that the outer diameter for either size core is identical. This outer dimension is relevant (see below) for fitting the collar that maintains a glove bag in place, if desired (Figs. 3D and 4). A small lip at the top of the stopper, the width of which is equal to the width of the core barrel (∼2 mm), holds the core in place as the sediment is extruded out the top of the barrel. This does not require compression of the barrel itself, as is typical of many other extruder designs. The combined core tube/stopper is then inserted through the top plate (Fig. 1- Item No. 1) of the main frame. and is locked into place using two adjustable brackets (Fig. 1- Item No. 12) just below the top plate.

A bottle jack (Fig. 1- Item No. 7) is placed on the baseplate (Fig. 1- Item No. 6) of the extruder, directly under the core, which is used to push the sediment through the top of the core barrel, stopping once the desired section thickness is exposed. We use spatulas to slice each extruded sediment section and transfer to a storage container (typically a 50 mL centrifuge tube) prior to processing, although some labs employ other techniques such as sliding sub-samplers [13]. The core stopper essentially creates a small platform that enables accumulation of the extruded sediment prior to collection. This is particularly useful when sampling unconsolidated near-surface sediment that tends to “flow” when extruded from the core barrel. “Soupy” sediment will collect on the surface created by the core stopper, rather than flowing down the sides of the core barrel, where it can be scooped up and placed into a sample container. When the maximum height of the jack has been reached, the piston is reset and push rods (Fig. 1- Item No. 32, Fig. 3C), constructed of 6-inch-long cylinders (2-inch diameter) fitted with metal dowels (Fig. 1- Item No. 33) and slots for easy assembly, are added below the piston. Note, at least one cylinder should not have a dowel inserted in either end to allow flush surface-to-surface contact between that cylinder and the piston or jack. This process is repeated until the entire sediment core is sectioned, or the target depth has been reached.

Some analytes of interest are subject to rapid oxidation when exposed to an oxic environment during sediment core processing, so the extrusion apparatus was designed for easy attachment of a glove bag in which an artificial anoxic environment can be maintained. Two plastic gaskets of equal size (Fig. 4- Item No. 26, 30) are joined together using neodymium magnets permanently affixed within the gaskets (Fig. 4- Item No. 27) to form a collar (Fig. 3D) that fits around the core stopper that protrudes from the top main frame plate (Fig. 3E). A circular hole of ∼5-cm diameter, between the inner and outer diameter of these gaskets, is cut in the base of the glove bag (Fig. 4 Item No. 31). One gasket is then placed inside the glove bag and the other outside with the neodymium magnetic inserts facing each other and the hole that was cut in the glove bag centered between them. The magnets hold the two gaskets together tightly and the rubber o-ring set within the bottom gasket (Fig. 4- Item No. 28) creates an airtight seal between the top and bottom of the collar. The gasket/glove bag assembly is placed around the core stopper, with an additional o-ring around the inner circumference of the bottom gasket (Fig. 4- Item No. 29), creating an airtight seal around the stopper itself. The glove bag can then be filled with N_2_ gas to prevent oxidation of the samples as the core is sectioned (Fig. 3F). Typically, the glove bag is purged of ambient air by closing all openings in the glove bag and filling with N_2_, then creating a small purge opening (in front glove bag opening flaps) and pushing all of the air out of the bag. This fill/purge process is repeated two additional times and the glovebag is permanently sealed on the fourth fill. Placing the centrifuge tubes into which sediment will be sampled during sectioning inside the glove bag and opening them slightly before purging ensures no O_2_ remains in the tubes during core processing. The airtight centrifuge tubes prevent oxygen from interfering with the sample post-sectioning, thus there is no need to eliminate headspace in the tubes by filling them completely, granting more flexibility with respect to obtaining thinner sediment sections.

## Benefits and method validation

This novel sediment core extrusion apparatus has multiple benefits compared to similar devices used in sediment research. With many other devices, the core barrel is attached to the extrusion apparatus with a clamp or adjustable collar, which holds the barrel in place while the sediment is extruded. The time it takes to process multiple cores is significantly longer using such a traditional tube compression setup than with the new apparatus described here. With the former technique, even slight compression of the barrel can prevent the extruder piston from traveling the full length of the tube, so the clamped core barrel must be removed and reclamped whenever the extruder piston reaches the clamp. In addition, the core barrel may slip vertically under heavy weight, if relying only on the squeezing of the outside of the tube to hold in place. Instead, in our extruder design, the lip at the top of the core stopper means there is no deformation of the barrel, so the entire core can be sectioned easily, without unclamping and repositioning the core barrel, and the stopper and barrel are easily separated so another core can be slid into place quickly.

Extrusion and sampling of sediment cores is often performed at or near the coring location to minimize sediment mixing and reduce the amount of time between core collection and sample preservation and analysis. Because our new sediment extrusion apparatus is a free-standing structure of adjustable height, it can be moved easily to different locations, does not need to be attached to a benchtop or table, can be assembled rapidly, and requires minimal space. This makes it optimal for sampling trips or cruises where space is limited and instruments need to be assembled and disassembled rapidly so other instruments can be set up as needed. The adjustable structure also enables users of all heights to use the apparatus comfortably, thereby limiting fatigue.

In addition to making the sectioning of cores easier and faster, this apparatus improves upon previous methods by preventing oxidation of redox-sensitive analytes. Although this is not the first apparatus designed to facilitate the use of a glove bag to create an artificial anoxic environment, it makes the use of such a bag simpler and cheaper. The magnetic collar used to attach the glove bag to the apparatus is unique as far as we know. It is easy to assemble, and creates a reliable air-tight seal around the core barrel, preventing the entry of oxygen into the environment inside the glove bag. The need to continually refill the glove bag to compensate for leaks is almost completely eliminated, and fewer gas tanks must be purchased and transported to the processing locations. Another benefit of this modification is that the surface area of the platform around the top of the core is increased, providing a larger area for material to amass while sectioning the core.

With respect to the fundamental technique, however, the new extruder system is no different from other glove bag implementations that have been extensively employed in the literature for decades. Regardless, two test demonstrations were conducted. In the first, a 0.1 M solution of Fe(NH4)_2_(SO_4_)_2_⋅6H_2_O was created in the prepared glove bag using N_2_-degassed water and serially diluted to obtain a triplicate set of 20 µM Fe^2+^ standards, each 10 mL in a 15 mL centrifuge tube (i.e. simulating pore waters separated as described below). The standards were left in the glove bag during the sectioning of a sediment core (∼90 min), and then a single aliquot from each tube was analyzed using the ferrozine method [14] (an Fe(II)-specific colorimetric technique) to ensure the Fe^2+^ was not lost to oxidation. The average recovery from the three measurements was 18.6 ± 1.7 µM, which may also include a small analytical error from pipetting as well.

For the next demonstration, vertical profiles of sediment pore water dissolved iron redox speciation from three sites in the hypereutrophic Lake Okeechobee, Florida, are presented to provide example data and further demonstrate the ability of the system to maintain redox speciation during core separation. Cores were sectioned using the new apparatus at 1 cm depth intervals inside an N_2_-filled glove bag to prevent oxidation. Porewaters were extracted from each section via centrifugation, filtered (0.22 µm PES) inside the glove bag, and immediately analyzed for dissolved reduced iron, Fe(II)_d_, using the ferrozine method [14]. Total dissolved Fe (Fe_d_) was also determined with the ferrozine method but after addition of a reductant (Hydroxylamine HCl) to an aliquot of porewater and reaction overnight to convert most Fe_d_ to Fe(II) [15]. The concentration of dissolved Fe(III) (Fe(III)_d_) was then calculated as the difference between total dissolved Fe and Fe(II)_d_.

The rationale for this selected redox pair is as follows. A pool of dissolved ferrous iron (“Fe(II)_d_”), which includes the aqueous Fe^2+^ ion as well as organic forms of Fe(II)), often accumulates in pore waters. Very often in freshwater, and routinely in marine systems, Fe(II)_d_ is often an indicator of the dissimilatory reduction of iron(III) (oxy)(hydr)oxide minerals (“Fe oxides”). This is a form of anerobic respiration coupled to the oxidation of reduced carbon substrates [4,16]. The Fe(II) is readily oxidized by aqueous O_2_ or anaerobic oxidants (NO_3_^−^, manganese oxides: MnO_2_, or even other Fe oxides) to precipitate as Fe(III) oxide minerals, or it can be stabilized in the dissolved form (“Fe(III)_d_”) as either soluble organic-Fe(III) complexes or Fe(III) colloids. On the other hand, the non-reductive, organic ligand-promoted solubilization of particulate Fe(III) oxides to form dissolved organically-complexed Fe(III)_d_ has also been demonstrated [16]. This may serve as a potentially biologically-mediated, non-oxidative intermediate step in the overall dissimilatory reduction of iron oxides. Indeed, Fe(III)_d_ formed from either pathway has been detected in numerous marine and estuarine environments [16], [17], [18], [19]. If Fe(II)_d_ is present in a pore water sample separated using the extruder system, then the detection of only low concentrations of Fe(III)_d_ would suggest that sample oxidation by O_2_ is limited, given the readiness by which Fe(II) undergoes aerobic oxidation. However, the detection of significant Fe(III)_d_ does not necessarily indicate that it has formed from artifactual oxidation.

**Fig. 5:**
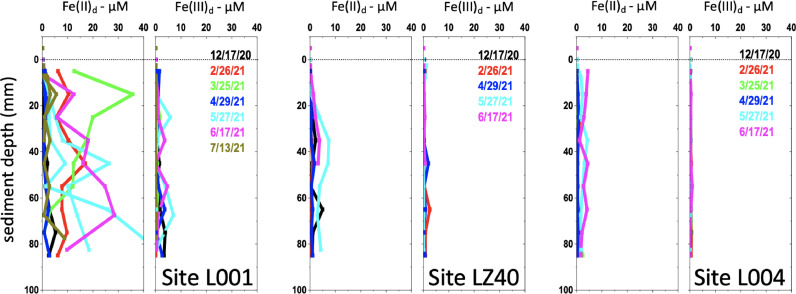
Lake Okeechobee core depth profiles of dissolved ferrous and ferric iron are presented to demonstrate that dissolved Fe(II) can be largely maintained in the reduced form in pore waters separated using the extruder. Site names correspond to those maintained by the South Florida Water Management District (www.sfwmd.gov). Site L001 is closer in proximity to the mouth of the Kissimmee River and is comprised of sand and mud, while sites LZ40 and L004 are instead accumulated mud in the center and east of the lake, respectively. Results from duplicate cores collected on 5/27/21 are presented from site L001. If lines/symbols are not visible, this is because measured concentrations are zero and profile data points are obscured by other profiles or cores were limited in depth.

Core site L001 is comprised of mixed sand and mud in the north of the lake and receives inflow and particulates deposited from the Kissimmee River. Sites LZ40 and L004 are in the deeper central and eastern areas of the lake, respectively, and have been fine-grained iron-rich sediment deposition centers for decades [20], fueled by anthropogenic perturbations to the watershed. Results show reactive solid phase iron hydroxides measured via ascorbic acid extraction decrease in the order L001 > LZ40 > L004 (Table 2). The ascorbic acid extraction retrieves the fraction that correlates with the potential for solid phase utilization by dissimilatory Fe-reducing microbes [21]. In turn, L001 contains the highest Fe(II)_d_ pore water concentrations (Fig. 5), although concentrations are relatively low in comparison to some other freshwater and estuarine sediments [16]. Still, if oxidation of porewater had occurred after core sectioning, Fe(III)_d_ would be expected to be present in significant concentrations. Fe(III)_d_ concentrations were averaged < 10% of total Fe_d_ at L001 [12]. Consistent with there being less solid phase iron hydroxides, the other sites displayed substantially less dissolved Fe, although a larger fraction, up to ~20%, was present as Fe(III)_d_. While this is not an insignificant amount, numerous studies from multiple research groups have demonstrated greater concentrations and proportions of Fe(III)_d_ relative to total Fe_d_ using similar glove bag systems (compiled in [16]). Overall, this dataset does demonstrate that the system (1) is capable of at a minimum maintaining the integrity of iron redox speciation as expected with a generic glove bag system; and (2) can be used to section core sediments for subsequent depth-resolved analyses with an improved efficiency relative to previous generation systems.

**Table 2:** Vertically and sampling-event averaged iron concentrations for the Lake Okeechobee sediment cores.

Site	Average reactive Fe hydroxides	Average pore water Fe(II)_d_	Average pore water Fe(III)_d_	Fe(III)_d_ relative to total dissolved Fe: (Fe(III)_d_ +Fe(II)_d_)
	µmol gram^-1^ of dry sediment	*μΜ*	*μΜ*	*%*
L001	76 ± 45	6.8 ± 8.6	0.74 ± 1.4	9.8 ± 17
LZ40	49 ± 17	1.3 ± 1.8	0.31 ± 0.49	19 ± 3.2
L004	47 ± 22	1.1 ± 1.3	0.16 ± 0.18	13 ± 1.9
